# Preliminary Study on Pain Reduction of Monosodium Iodoacetate-Induced Knee Osteoarthritis in Rats by Carbon Dioxide Laser Moxibustion

**DOI:** 10.1155/2014/754304

**Published:** 2014-06-12

**Authors:** Fan Wu, Ruixing Zhang, Xueyong Shen, Lixing Lao

**Affiliations:** ^1^Shanghai University of Traditional Chinese Medicine, Shanghai 201203, China; ^2^Center for Integrative Medicine, School of Medicine, University of Maryland, Baltimore, MD 21201, USA; ^3^School of Chinese Medicine, The University of Hong Kong, Hong Kong

## Abstract

In order to study the effects of CO_2_ laser moxibustion on the pain and inflammatory cytokine expression in the spinal dorsal horn of rats with monosodium iodoacetate- (MIA-) induced knee osteoarthritis (KOA), we designed an experiment by randomly assigning 8 SD rats into 3 groups, namely, a CO_2_ laser moxibustion group, a sham treatment group, and a blank control group. The treatment group received a laser moxibustion on acupoint Dubi (ST 35; 5 min/treatment, 1 treatment/day) for 8 days, and after treatment, the rats exhibited significantly increased interhindpaw differences compared with their preinduction values. Meanwhile, cytokine microarray analysis showed that one cytokine (TIMP-1) was significantly upregulated and two cytokines (Agrin and MMP-8) were significantly downregulated in treatment group. The present study suggested that CO_2_ laser moxibustion created certain pain reduction in the rats with MIA-induced KOA and significantly inhibited the expression of most inflammatory cytokines in the ipsilateral spinal dorsal horn.

## 1. Introduction

Osteoarthritis (OA) is the most common joint disease in adults [1], prevalently affecting the knee [2]. The major clinical presentation of knee OA is a chronic pain of the affected knee. Current treatment of OA primarily aims at reducing the pain. However, a meta-analysis found that, for reducing short-term pain in OA, commonly used drugs (NSAIDs and nonsteroidal anti-inflammatory drugs) performed only slightly better than placebos [3].

Pain reduction with acupuncture and moxibustion has been a focus in recent years. But these researches mainly concentrated in the field of needling analgesia, whereas little attention has been given to the moxibustion. Although both stimulations target acupoints, needling and moxibustion deliver different modes of actions to acupoints, thereby likely acting via different mechanisms.

Studies on KOA have demonstrated high expression of multiple inflammatory cytokines in synovial fluid, synovium, joint capsular tissue, and cartilage from OA patients with severe pain [4]. These cytokines are believed to further stimulate the function expression of other inflammatory mediators, eventually causing pain [5, 6]. Im et al. [7] proposed that the mechanism of pain in MIA-induced rat knee OA was similar to that in the animal model of neuropathic pain, suggesting a potential overlap in their conduction pathways. A similar situation has been found in a rat model of medial meniscus transection [8].

In the present study, we induced KOA in rat with MIA and treated the acupoint Dubi (ST 35; the point most commonly targeted in needling treatment of knee OA) with carbon dioxide (CO_2_) laser simulating the thermal and infrared irradiation effects of moxibustion. Our objective was to investigate whether the CO_2_ laser treatment could (1) reduce pain in this model and (2) affect the expression of inflammatory cytokines in the spinal dorsal horn.

## 2. Materials and Methods

### 2.1. Animals

Eight male Sprague Dawley rats weighing 200–220 g (Harlan, USA) were kept under controlled laboratory conditions (22 ± 0.5°C, relative humidity 40%–60%, 12 h alternate light-dark cycles, and food and water ad libitum). Each rat was given a 4-week training for adaptation to the laboratory environment, hindpaw weight-bearing tests, and simulated treatments. The training was conducted daily at regular times. After the training, the rats weighted 250–275 g.

### 2.2. Induction of Knee OA

The rats were randomly assigned to a CO_2_ laser moxibustion group (*n* = 3), sham treatment group (*n* = 3), and blank control group (*n* = 2). For induction of osteoarthritis with MIA, rats from laser treatment group and sham treatment group were anesthetized with isoflurane (Piramal Critical Care, USA) in oxygen and administered a single percutaneous intra-articular injection of 1.0 mg of MIA (Sigma, USA) through the infrapatellar ligament of the left knee. MIA was dissolved in 0.9% saline and administered in a volume of 0.05 mL using a 26 gauge, 0.5 inch needle [9].

### 2.3. Measurement of Hindpaw Weight-Bearing Distribution

After OA induction, the original balance in weight-bearing capability of hindpaws was disrupted. An incapacitance meter tester (Model-600R, IITC Life Science, USA) was employed for determination of hindpaw weight distribution. Rats were placed in an angled plexiglass chamber positioned so that each hindpaw rested on a separate force plate. The force exerted by each hindlimb (measured in grams) was averaged over a 10 s period. Each data point is the mean of six, 10 s readings. The change in hindpaw weight distribution was calculated by determining the difference in the amount of weight (g) between the left and right limbs.

### 2.4. Treatment after KOA Induction

From 1 day after KOA induction, the CO_2_ laser moxibustion group underwent laser treatment on the depression of the lateral aspect of the infrapatellar ligament (the acupoint is equivalent to ST-35 in human) for 8 days (one treatment per day). The rat was placed on a platform, and the head was covered by the operator's hand. Then the hindlimb was extended to expose the acupoint. After the animal became calm, the acupoint was irradiated with a CO_2_ laser beam for 5 min. The wavelength of CO_2_ laser is 10.6 *μ*m and the output power density is 63.29 mW/mm^2^. The rat of the sham treatment group was similarly immobilized on the platform without undergoing the laser treatment.

### 2.5. Microarray Analysis of Cytokines

Twenty days after the completion of laser (or sham) treatment, all animals were anesthetized by intraperitoneal injection of pentobarbital (60 mg/kg) and killed by decapitation. The Entire spinal cord was ejected, and the dorsal horn was dissected. The dorsal horn was homogenized in protein extraction buffer containing 1% EDTA and 1% Halt protease and phosphatase inhibitor cocktail (Thermo Scientific, USA). After centrifugation (14,000 rpm for 10 min at 4°C), the supernatant containing the proteins was collected. Protein concentration was determined with Bio-Rad protein assay kits (Bio-Rad, USA). Cytokine levels in the dorsal horn were analyzed with cytokine antibody microarrays (Rat Cytokine Array C2, RayBio, USA) following the guideline by manufactory. After development and scan, for each sample, membranes with optimal image qualities (high signal/noise ratio, well-defined background, and absence of signal overlap with adjacent dots) were analyzed using Image J (National Institute of Health, USA) equipped with a protein array analyzer add-on. In image analysis, C1, C2, D1, and D2 served as the background (Table 1) and A1, A2, L7, and L8 as the positive reference. Finally, results recorded from each membrane were normalized.

### 2.6. Statistical Analysis

Results are expressed as the mean ± SD. Results of repetitive testing of hindpaw weight distribution were analyzed using a repeated-measures general linear model. The mean changes in hindpaw weight distribution over time for the laser treatment group were compared with the mean change in hindpaw weight distribution over time in the sham treatment group via the Bonferroni multiple comparisons procedure. The signal densities of cytokines were analyzed using a multivariate analysis of variance with Bonferroni post hoc test. *P* values less than 0.05 were considered significant.

## 3. Results

### 3.1. Changes in Hindpaw Weight-Bearing Distribution

**Figure 1:**
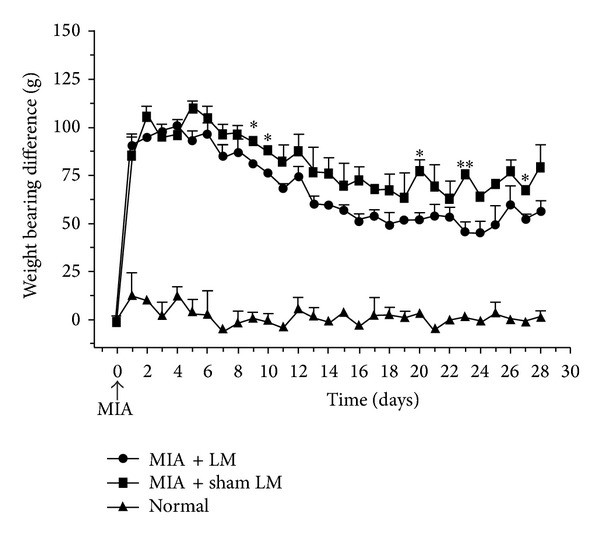
Variation of interhindpaw weight-bearing difference after induction of knee osteoarthritis (MIA + LM: CO_2_ laser moxibustion group; MIA + sham LM: sham treatment group; Normal: blank control group;  *P < 0.05,  **P < 0.01). Values are the mean and SD.

Before OA induction (Figure 1), the three groups exhibited similar interhindpaw weight-bearing distributions (*P* > 0.05). Throughout the study, the value recorded from the blank control group varied slightly (*P* > 0.05). One day after OA induction, the values recorded from the laser moxibustion group and sham treatment groups increased significantly compared with their baseline values (both *P* < 0.001). And the laser moxibustion group and sham treatment group were similar at this time point. Values measured from the laser moxibustion group and sham treatment group both decreased with time (both *P* < 0.05), but at five time points the laser moxibustion group showed better improvement than the sham treatment group: 1st day (*P* = 0.044), 2nd day (0.029), 12th day (0.031), 15th day (0.007), and 19th day (0.016) after the completion of all the treatment.

### 3.2. Cytokine Levels

**Figure 2:**
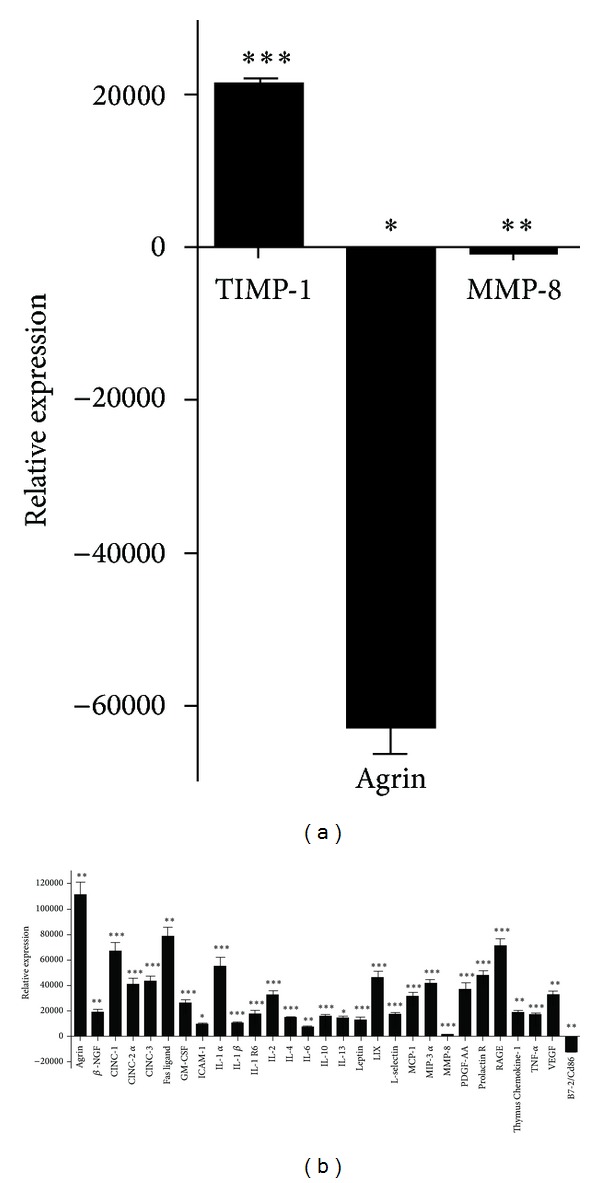
Relative expression levels of cytokine in left dorsal horn tissues measured from the (a) CO_2_ laser moxibustion group and (b) sham treatment group 28 d after induction of knee osteoarthritis (results of microarray analyses; upward bars indicate upregulation versus blank control; downward bars indicate downregulation; MIA + LM: CO_2_ laser moxibustion group; MIA + sham LM: sham treatment group; Normal: blank control group;  *P < 0.05, **P < 0.01, and  ***P < 0.001). Values are the mean and SD. Beta-NGF: beta-nerve growth factor; CINC-1/2 alpha/3: cytokine-induced neutrophil chemoattractant-1/2 alpha/3; GM-CSF: granulocyte-macrophage colony-stimulating factor; ICAM-1: intercellular adhesion molecule 1; IL-1 alpha/1 beta/1 R6/2/4/6/10/13: interleukin-1 alpha/1 beta/1 R6/2/4/6/10/13; LIX: lipopolysaccharide induced CXC chemokine; MCP-1: monocyte chemotactic protein-1; MIP-3 alpha: macrophage inflammatory protein-3 alpha; MMP-8: metalloproteinases 8; PDGF-AA: platelet-derived growth factor-AA; RAGE: receptor for advanced glycosylation end products; TNF-alpha: tumor necrosis factors-alpha; VEGF: vascular endothelial growth factor.

The expression of inflammatory cytokines in the dorsal horn samples was analyzed with RayBio Rat Cytokine Array C2 microarrays, which allowed simultaneous detection of 34 inflammation-related cytokines. In the laser moxibustion group, signal densities of most cytokines were similar or lower than corresponding values recorded in the blank control group (data not shown). Compared with the control group (Figure 2(a)), the signal densities of Agrin (*P* = 0.036) and MMP-8 (*P* = 0.006) were significantly downregulated, and only one cytokine (TIMP-1) was significantly upregulated (*P* < 0.001). In the sham treatment group (Figure 2(b)), the signal densities of most inflammatory cytokines were upregulated, including ICAM-1, IL-13 (both *P* < 0.05), Agrin, beta-NGF, Fas Ligand, IL-6, Thymus Chemokine-1, VEGF-A (all *P* < 0.01), CINC-1, CINC-2 alpha, CINC-3, GM-CSF, IL-1 alpha, IL-1 beta, IL-1 R6, IL-2, IL-4, IL-10, Leptin, LIX, L-Selectin, MCP-1, MIP-3 alpha, MMP-8, PDGF-AA, Prolactin R, RAGE, and TNF alpha (all *P* < 0.001). In comparison, only one cytokine (B7-2/CD86) was downregulated (*P* < 0.05).

## 4. Discussion

### 4.1. Understanding of Knee OA from the Perspective of Traditional Chinese Medicine (TCM)

In the TCM theory, knee OA is categorized as a* Bi* syndrome and frequently diagnosed as cold arthralgia (*Han Bi*) or pain arthralgia (*Tong Bi*). Consequently, in TCM practices, this disorder is commonly treated by moxibustion. In the present study, the lateral depression on the infrapatellar ligament (analogous to acupoint Dubi ST-35 in human) of the affected knee was treated by CO_2_ laser moxibustion. According to the acupuncture and moxibustion theory and because of the anatomic proximity of this acupoint to the lesion, moxibustion on this point created a warming effect on the local meridian* Qi and Blood* circulation, thereby reducing pathogenic factors.

### 4.2. Rational of Laser Treatment

Traditionally, the therapeutic effects of moxibustion have been attributed to the thermal action. Our earlier studies found that, besides the thermal action, infrared (IR) radiation is another potential therapeutic mechanism underlying the moxibustion treatment. We observed that, during indirect moxibustion, the infrared radiation from the burning moxa stick was 20 times higher than the self-IR radiation from human acupoints in energy intensity, but the spectral profiles of the two radiation waves were similar [10]. This finding suggests that infrared radiation may be another important therapeutic mechanism in indirect moxibustion. To test this assumption, here we adopted CO_2_ laser moxibustion as a substitute to the traditional indirect moxibustion. The laser treatment may have several advantages. First, with the principal wavelength bands centering around 10.6 *μ*m, the CO_2_ laser is easily absorbed by water molecules in the skin and generates heat. By adjusting the laser power, it is possible to control the heating effect and skin temperature. Second, the laser wavelength is close to the peak of self-IR radiation from human acupoints, similar to the traditional indirect moxibustion [11]. Additionally, the use of CO_2_ laser moxibustion can eliminate confounding factors in traditional indirect moxibustion, such as smokes.

If the CO_2_ laser moxibustion can provide similar effects to traditional indirect moxibustion, it may also offer multiple benefits for better understanding of therapeutic mechanisms of moxibustion. First, the output power of the laser irradiation can be adjusted, thereby allowing determining the effects of different temperatures on acupoints. Second, by controlling the thermal actions, the effects of infrared radiation on acupoints can be directly studied. Finally, even if the laser moxibustion fails to achieve equivalent effects of the traditional moxibustion, it suggests the presence of other unknown mechanisms underlying moxibustion therapies.

### 4.3. Pain Reduction of Laser Treatment

MIA induces OA via inhibiting glycolysis and, thus, causing chondrocyte death [12]. In the present study, 1 mg of MIA was injected into the left knee to induce OA. One day after OA induction, the rats exhibited clear changes in hindpaw weight-bearing distribution (i.e., preferentially putting more weight load on the right hindpaw). During subsequent 7-day laser (or sham) treatment and 20-day laboratory monitoring, the laser moxibustion group and sham treatment group showed gradual decreases in interhindpaw difference (Figure 1). Although the laser moxibustion group seemed to have a greater decrease than did the sham treatment group, the difference between the groups was not statistically significant. This may have been attributed to the small sample sizes used in this study. A calculation using means and standard deviations recorded from the two groups 1 day after the completion of laser (or sham) treatment suggested that minimally 6 animals are required per group for the recognition of the statistical difference (assuming *α* = 0.05, 1 − *β* = 90%, equal group sizes). A calculation using data recorded at the end of this study suggested that 7 animals are required per group (assumptions the same as previous calculation).

### 4.4. Cytokine Expression in Spinal Dorsal Horn

The pain mechanisms in OA are not fully understood. Inflammatory cytokines in the joint region are categorized into two groups: pro- and anti-inflammatory cytokines [13]. Main proinflammatory cytokines include IL-1 alpha/beta, TNF-alpha, IL-6, IL-8, IL-17, and IL-18. Anti-inflammatory cytokines mainly include IL-4, IL-10, IL-11, IL-13, IL-1Ra, and IFN-gamma. However, despite the presence of these numerous cytokines, OA is not considered an inflammatory disorder. Because of the absence of nerves in the joint cartilage [14], cartilage degradation cannot directly cause pain. In the knee OA model used in the present study, chondrocytes at the lesion site produced chemokines, cytokines, and proteinases. These molecules sensitized endings of primary afferent fibers in the adjacent tissues [15]. With increasing signal input from nociceptors in the OA site to the spinal neurons, the neurons became more sensitive to signal from the knee [16]. With the increase of spinal neuron excitability, the sensitive threshold of neurons in the spinal dorsal horn to peripheral injurious stimuli decreased [17].

Current studies on the mechanisms of pain development are primarily focused on spinal glial cells [18]. After the occurrence of peripheral tissue injuries, these cells (including microglial cells and astrocytes) respond by activities such as antigen presentation (primarily microglial cells) and release of inflammatory mediators. These responses may be a cause of the chronic pain in OA [19, 20]. Studies have confirmed that, in animals with MIA-induced knee OA, the proliferation of microglial cells in the ipsilateral spinal dorsal horn is associated with the development of neuropathic pain [21]. In the sham treatment group of the present study, the levels of most analyzed proinflammatory cytokines in the ipsilateral spinal dorsal horn increased significantly compared with the blank control (Figure 2(b)), consistent with earlier findings [22]. This indicated that nociceptive stimulus in the lesion site was transferred into the spinal cord and generated a large number of inflammatory cytokines, probably via pathways involving spinal neurons or glial cells. In comparison, in the laser moxibustion group (Figure 2(a)), only three inflammatory cytokines varied significantly compared with the blank control group. Notably, one cytokine (TIMP-1) significantly upregulated in the laser moxibustion group was not significantly changed in the sham treatment group, and two cytokines (Agrin and MMP-8) significantly downregulated in the laser treatment group were significantly upregulated in the sham treatment group. Of these “oppositely changing” cytokines, MMP-8 and TIMP-1 may deserve particular attention. MMP-8 is a member of the matrix metalloproteinase (MMP) family and believed to be closely related to OA-induced cartilage destruction, as confirmed by studies on humans and animal models [23, 24]. TIMP-1 (tissue inhibitors of metalloproteinases 1) is a MMPs inhibitor and serves to regulate the activities of MMPs. MMPs and TIMP-1 function collaboratively to maintain the homeostasis of the extracellular matrix. Studies have confirmed the roles of the two cytokines in the peripheral and central nervous of model animals with OA. Janusz et al. found that oral administration of TIMP-1 significantly alleviated cartilage destruction in rats with MIA-induced knee OA [25]. Several studies reported that peripheral nerve injuries stimulated upregulation of MMP-9 and MMP-2 in the spinal cord [26] and subsequently activated microglial cells and astrocytes, thereby leading to neuropathic pain [27–29]. Additionally, Kawasaki et al. observed that intrathecal injection of TIMPs significantly reduced the neuropathic pain [26]. The mechanisms of neuropathic pain following peripheral nerve injury-induced glial cell activation in the dorsal horn are not fully understood. However, relevant results suggested that TRPV1 (transient receptor potential cation channel subfamily V member 1) may be related to neuropathic pain involving MMPs [30]. It has been shown that TRPV1 is activated at ≥43°C [31]. In the present study, the laser treatment on the acupoint created a local temperature around 45°C. Accordingly, we suggest the following processes as a potential mechanism for explaining the effects of laser treatment on spinal cytokine expression. After the MIA induction of knee OA, inflammatory events occurred in the joint cartilage and synovium. Inflammatory mediators stimulated local nociceptors, leading to sensitization of primary afferent fibers and then central sensitization. Spinal neurons and glial cells in the ipsilateral dorsal horn were activated, thus releasing a variety of inflammatory cytokines. The CO_2_ laser treatment on acupoint Dubi activated TRPV1 channels in the irradiated region. TRPV1 probably interacted with various inflammatory cytokines [32] and, thereby, modulated their expression. Of these cytokines, the modulation of MMPs may have produced important roles. Presumably, TRPV1 expression propagated from primary afferent fibers to the dorsal root ganglion and inhibited MMPs expression in the ganglion. Additionally, TRPV1 may have upregulated TIMPs in the dorsal horn and further inhibited their expression there. These functions suppressed the activation of spinal glial cells, thereby providing pain reduction. The acupoint functioned analogous to an amplifier, spreading limited stimuli to a larger space. This hypothetical explanation, however, should be verified by further studies.

As a preliminary work, this study has several limitations. First, the sample size was too small to allow statistical interpretation of behavioral changes. Second, the local affected region (i.e., joint cartilage, subchondral bone, and synovium) was not analyzed by microarray analysis. Consequently, the effects of CO_2_ laser moxibustion on the local expression of inflammation-related cytokines could not be determined. Finally, the study only attempted a preliminary screening of inflammatory cytokines in the dorsal horn after CO_2_ laser moxibustion. More qualitative and quantitative works are required for the determination of cytokines underlying the effects of CO_2_ laser moxibustion and their functional pathways.

## Figures and Tables

**Table 1:** Illustration of cytokines distribution as given by the Rat Cytokine Array C2 microarray (image provided by RayBio).

	A	B	C	D	E	F	G	H	I	J	K	L
1	POS	POS	NEG	NEG	Activin A	Agrin	B7-2/Cd86	beta-NGF	CINC-1	CINC-2 alpha	CINC-3	CNTF
2	POS	POS	NEG	NEG	Activin A	Agrin	B7-2/Cd87	beta-NGF	CINC-2	CINC-3 alpha	CINC-4	CNTF
3	Fas Ligand	Fractalkine	GM-CSF	ICAM-1	IFN-gamma	IL-1 alpha	IL-1 beta	IL-1 R6	IL-2	IL-4	IL-6	IL-10
4	Fas Ligand	Fractalkine	GM-CSF	ICAM-2	IFN-gamma	IL-2 alpha	IL-2 beta	IL-1 R7	IL-2	IL-4	IL-6	IL-10
5	IL-13	Leptin	LIX	L-Selectin	MCP-1	MIP-3 alpha	MMP-8	PDGF-AA	Prolactin R	RAGE	Thymus Chemokine-1	TIMP-1
6	IL-14	Leptin	LIX	L-Selectin	MCP-2	MIP-4 alpha	MMP-9	PDGF-AA	Prolactin R	RAGE	Thymus Chemokine-2	TIMP-2
7	TNF-alpha	VEGF	Blank	Blank	Blank	Blank	Blank	Blank	Blank	Blank	Blank	POS
8	TNF-alpha	VEGF	Blank	Blank	Blank	Blank	Blank	Blank	Blank	Blank	Blank	POS
